# Supplementary material for: Causal interplay between lactose intolerance and gut microbiota: a combined bidirectional Mendelian randomization and in vivo validation study
Source: Front Nutr. 2026 Jun 1;13:1803337. doi: 10.3389/fnut.2026.1803337 (PMC13265576; doi:10.3389/fnut.2026.1803337)
Supplement: Supplementary file 1 [file Data_Sheet_1.zip › supplementary materials/Forward/leave one out cross validation/ebi-a-GCST90027449.finngen_R12_E4_LACTONAS.pdf]

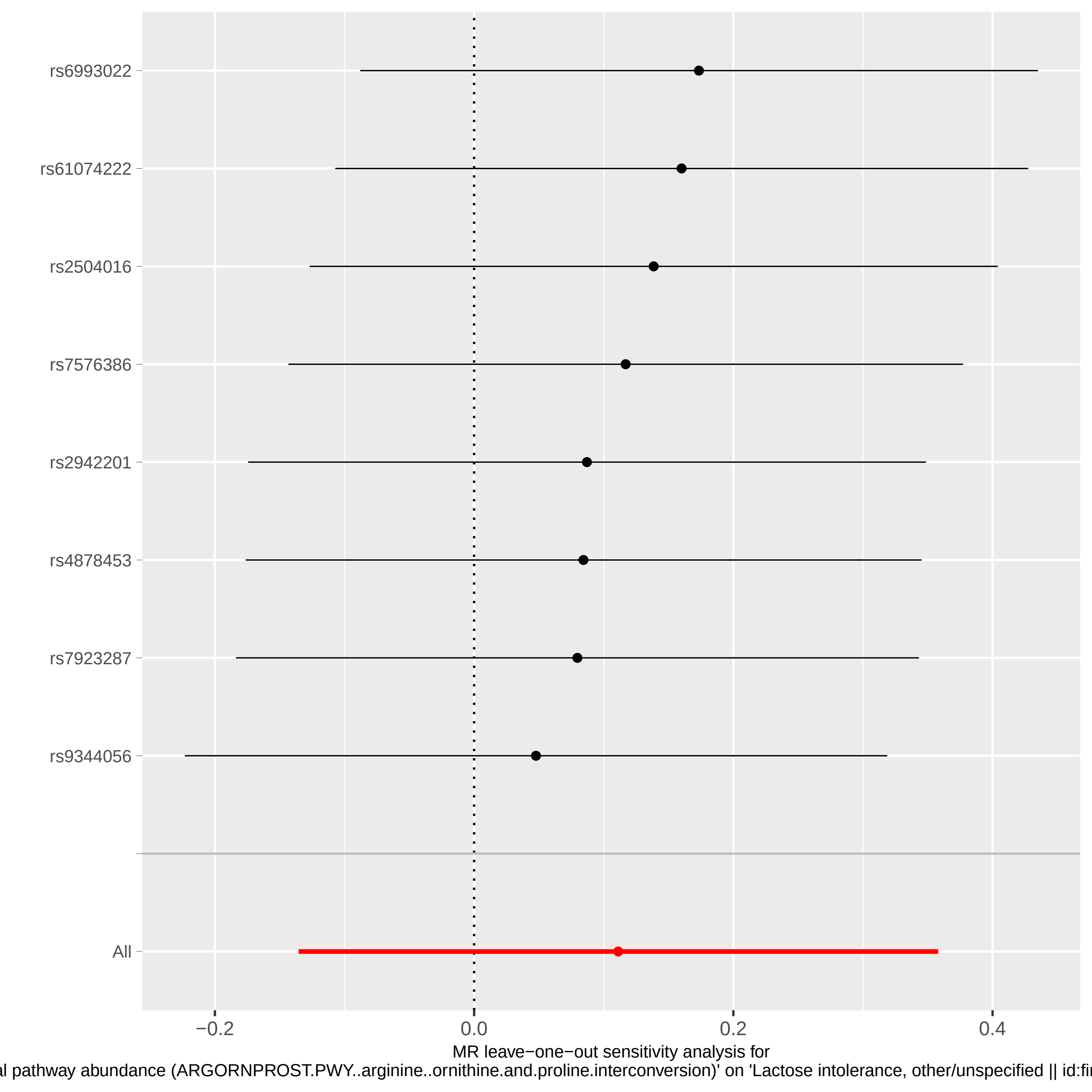

rs6993022
rs61074222
rs2504016
rs7576386
rs2942201
rs4878453
rs7923287
rs9344056
All
−0.2
0.0
0.2
0.4
MR leave−one−out sensitivity analysis for
al pathway abundance (ARGORNPROST.PWY..arginine..ornithine.and.proline.interconversion)' on 'Lactose intolerance, other/unspecified || id:fi
